# Hepatic Adenomatosis in a Transgender Man on Gender-Affirming Testosterone Therapy

**DOI:** 10.14309/crj.0000000000001483

**Published:** 2024-09-11

**Authors:** Yuting Huang, Nicole M. Loo, Alice Y. Chang, Zachary Yu, Amanda L. McKenna, Charles Ritchie, Allie M. Metcalfe, Raouf E. Nakhleh, Murli Krishna, C. Burcin Taner, Liu Yang

**Affiliations:** 1 Division of Gastroenterology and Hepatology, Department of Internal Medicine, Mayo Clinic, Jacksonville, FL; 2 Department of Transplantation, Mayo Clinic, Jacksonville, FL; 3 Division of Endocrinology, Diabetes, Metabolism and Nutrition, Department of Internal Medicine, Mayo Clinic, Jacksonville, FL; 4 Department of Radiology, Mayo Clinic, Jacksonville, FL; 5 Department of Pathology, Mayo Clinic, Jacksonville, FL

**Keywords:** hepatic adenomatosis, transgender, gender-affirming hormone therapy, testosterone

## Abstract

The management of hepatic adenoma in transgender individuals undergoing gender-affirming hormone therapy remains unclear, especially whether treatment should be based on sex assigned at birth or therapy patient received. We presented a transgender man, female at birth, with hepatic adenomatosis with molecular profile differed from typical adenomas in cisgender males on testosterone. Discontinuing testosterone led to autoinfarction of the adenoma, allowing the avoidance of invasive treatments and resumption of gender-affirming hormone therapy. This case underscores the necessity for personalized care in the growing transgender population and challenges current consensus of treatment based on sex assigned at birth, emphasizing a tailored approach.

## INTRODUCTION

There are 25 million transgender individuals globally, counting <1% of total population, while affecting younger generations disproportionally (1.43% in age 13–17 population).^1,2^ Depression, suicidality, and anxiety among transgender and gender diverse (TGD) individuals can be related to dysphoria or gender incongruence.^3^ Gender-affirming hormone therapy (GAHT) is critical in treating gender dysphoria and significantly enhances health outcomes.^4^

Sexual hormone use in cisgender population, including oral contraception pills and exogenous androgen mostly for body building, usually has higher doses and increases risk of hepatic adenomas.^5^ Compared to adenomas developed in women on estrogens, those that develop in men on androgens have a higher risk of malignant transformation and require aggressive management.^6,7^ GAHT, on the contrary, targets physiological hormone levels, and its risk for adenoma is unknown.^5^ Data on hepatic adenoma in TGD individuals on GAHT are scarce, with only 3 cases in transgender men, including 2 cases using oral 17-alkylated anabolic steroids, which are not recommended by guidelines due to hepatotoxicity.^4,8-10^ The only case reported using parenteral testosterone^11^ was treated with segmentectomy, surgical radiofrequency ablation, and transcatheter arterial chemoembolization, due to positive β-catenin staining, which increases the risk for malignancy.

## CASE REPORT

**Figure 1.**
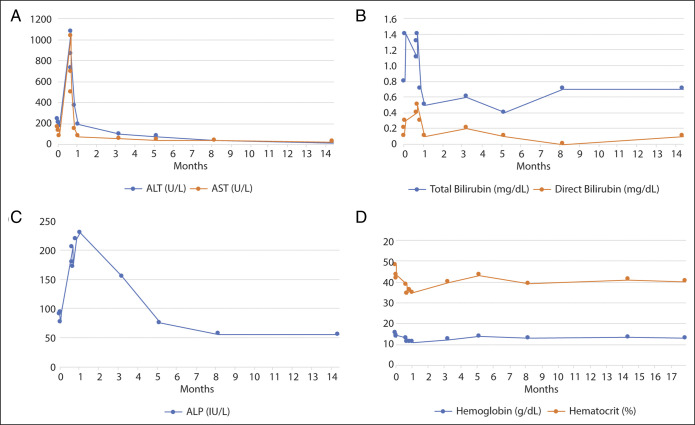
Laboratory tests throughout the disease course. (A) Aspartate transaminase (AST) and alanine transaminase (ALT) and (B) total bilirubin and direct bilirubin peaked during the initial presentation then normalized. (C) Alkaline phosphatase peaked 1 week after AST and ALT levels and gradually declined. All liver function tests returned to normal range 8 months after the initial presentation. (D) Hemoglobin and hematocrit initially dropped upon presentation due to intra-adenoma hemorrhage, but returned to normal levels. Resumption of topical testosterone treatment did not result in elevated hemoglobin or hematocrit levels.

**Figure 2.**
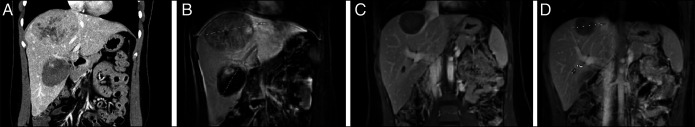
Radiological imagine studies of hepatic adenoma. (A) Initial computed tomography (CT) revealed a dominant liver mass in segment VIII with necrosis and heterogeneous enhancement, along with a 6.4 × 6.1-cm lesion in segment V and multiple smaller bi-lobar arterially enhancing lesions. (B) Initial magnetic resonance (MR) imaging upon presentation showed a 10.1 × 9.6-cm heterogeneously enhancing hypervascular and necrotic mass in hepatic segment VIII and a 6.9 × 6.4-cm mass in hepatic segment V. (C) Follow-up MR at 17 months postinitial presentation, before testosterone reinitiation, demonstrated a stable 4.8-cm nonenhancing defect in segment VIII and a stable 0.9-cm nonenhancing defect in segment V, likely reflecting autoinfarction. (D) Follow-up MR at 21 months postinitial presentation, 4 months after testosterone reinitiation, showed a stable 3.8-cm nonenhancing defect in segment VIII and a stable 0.9-cm nonenhancing defect in segment V with no interval growth in either lesion.

**Figure 3.**
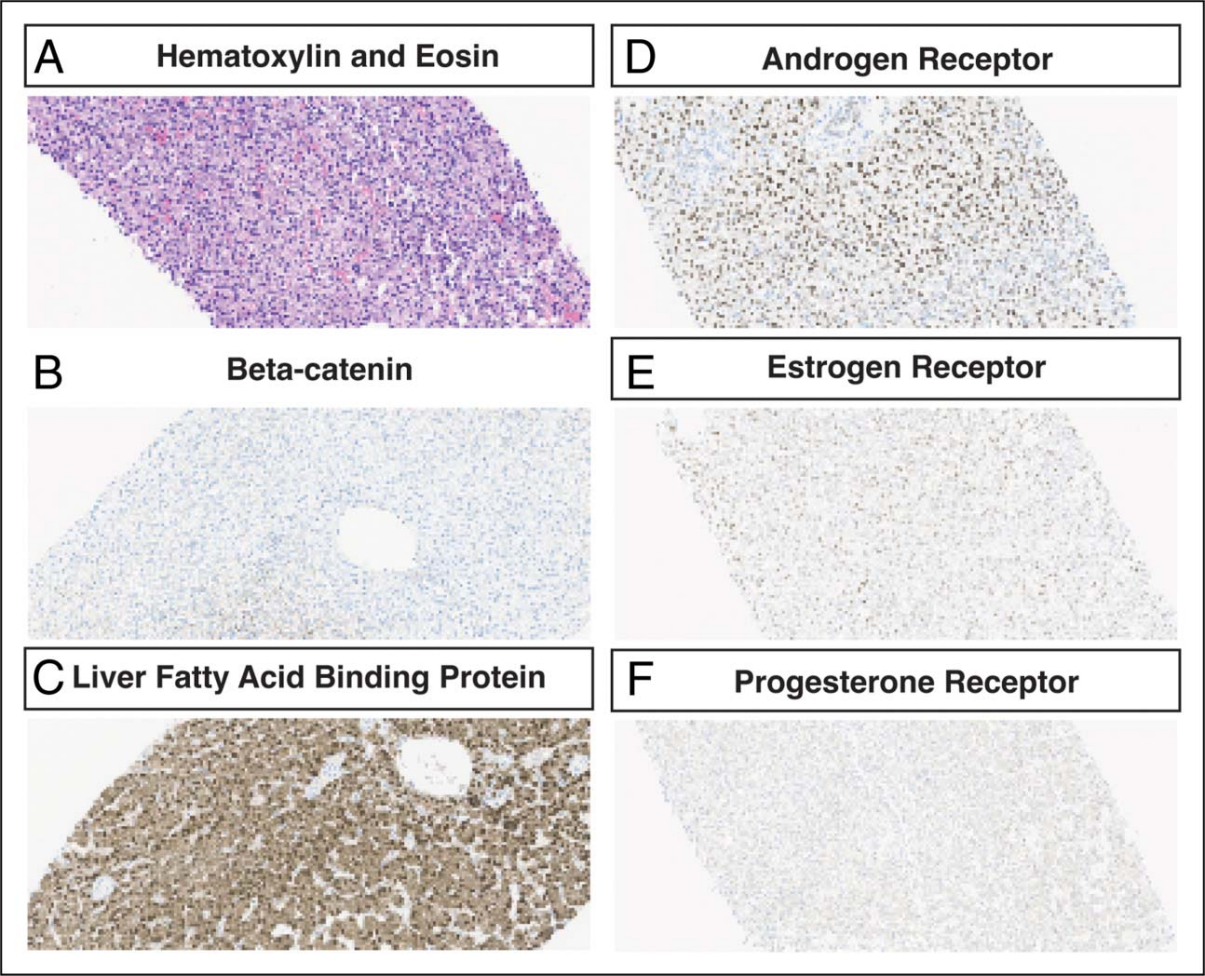
Pathology analysis of molecular subtype of hepatic adenoma. Hematoxylin and eosin staining revealed typical pathology findings of hepatic adenoma, characterized by sheets of cells with mild variation in cell and nuclear size, absence of bile ducts or portal tracts, and no cirrhosis (A). Immunohistochemistry staining showed beta-catenin negative (B), diffusely positive for liver fatty acid binding protein (C), positive staining for androgen receptor (D), weak positivity for estrogen receptor (E), and negativity for progesterone receptor (F) (magnification 20×).

A 22-year-old transgender man (body mass index 21.3 kg/m^2^) with no liver disease history and normal baseline liver function tests presented with right upper quadrant pain. At age 17, he started GAHT with weekly intramuscular injections of testosterone cypionate. He also took norethindrone, an oral progestin-only contraceptive, to suppress uterine bleeding. At age 18, he underwent a masculinizing bilateral mastectomy. He had normal vital signs, hepatomegaly, and right upper quadrant tenderness without guarding or rebound. Lab results showed elevated aspartate aminotransferase (1,033 U/L), alanine aminotransferase (1,071 U/L), and alkaline phosphatase (205 U/L) (Figure 1). Computed tomography showed a dominant liver mass centered in segment VIII with necrosis and heterogeneous enhancement, and a 6.4 × 6.1-cm lesion in segment V, with multiple additional smaller bi-lobar arterially enhancing lesions (Figure 2). Magnetic resonance imaging (MRI) detected over 15 bi-lobar masses, the largest 2 measured 10.1 and 6.9 cm, with intra-adenoma bleeding and necrosis (Figure 2). Exogenous testosterone and progesterone were discontinued. Pathology from fine needle aspiration of the dominant mass showed hepatic adenoma (Figure 3). Immunohistochemistry staining revealed negativity for β-catenin, ruling out β-catenin hepatic adenoma, and positivity for glutamine synthetase, liver fatty acid binding protein, and C-reactive protein, ruling out inflammatory and hepatocyte nuclear factor-1 alpha hepatic adenomas (Figure 3).^6^ The hormone receptor profile exhibited estrogen receptor ++, progesterone receptor −, and androgen receptor +++ (Figure 3). Since immunohistochemistry profiling showed low risk for malignancy transformation, a conservative treatment was pursued with regular hepatology clinic follow-ups and serial image studies. His liver function returned to normal 7 months later, and hepatic adenomas gradually reduced in both size and quantity (Figure 1). Seventeen months after presentation, the patient reported persistent dysphoria and uterine bleeding. Low-dose transdermal testosterone was started considering its lower risk of adenoma recurrence.^12^ Baseline MRI before restarting testosterone showed 2 stable nonenhancing defects (4.8 and 0.9 cm) (Figure 2). One month after reinitiating GAHT, the patient's testosterone levels reached therapeutic ranges (total testosterone 317 ng/dL [240–950 ng/dL], free serum testosterone 10.3 ng/dL [5.25–20.7 ng/dL]). Four months after reinitiating GAHT, the patient was free of symptoms. He had normal liver function and testosterone levels. Repeat MRI showed no interval changes of the 2 dominant lesions (3.8 and 0.9 cm), with no new lesions developed (Figure 2).

## DISCUSSION

We presented a case of hepatic adenomatosis complicated by necrosis and hemorrhage in a transgender man assigned female at birth, who was on high-dose testosterone injections. The condition was managed with observation. GAHT was reinitiated with low-dose transdermal testosterone therapy.

The identification of genetic alterations and deregulated signaling pathways revolutionized the classification and treatment of hepatic adenoma.^6^ Estrogen exposure have been associated with inflammatory and Sonic Hedgehog hepatic adenomas. Androgen use and male gender are often related to catenin beta 1-mutated hepatic adenomas, which have a higher risk of malignant transformation and warrant more aggressive treatments, including surgery or interventional radiology treatment.^6,7^ Hepatic adenomatosis (≥10 adenomas) has an even higher risk of spontaneous rupture, hemorrhage, and malignant transformation.

In our case, hepatic adenomatosis presented with both high-risk features (multiple lesions and large size) and low-risk features (absent of catenin beta 1 mutation, lack of nuclear atypia, and a normal nuclear ratio) for malignant transformation. The adenomas had autoinfarction and bleeding, a process that intensified after discontinuation of GAHT. The hemorrhage can be contributed by the considerable tumor load and hormone withdrawal. Typically, Sonic Hedgehog adenoma presents with hemorrhage clinically, but it has strong estrogen receptor expression and is more commonly seen in obese patients, not consistent with this case.^6,7^ Notably, after discontinuing GAHT, the decrease in sex hormone levels is almost 10-fold more drastic in a transgender man (testosterone levels drop from normal male range [240–950 ng/dL] to the normal female range [8–60 ng/dL]), compared to a transgender woman (estradiol concentrations drop from the normal female range [100–200 pg/mL] to the normal male range [10–40 pg/mL]).^6^ This rapid fall in testosterone concentrations with significant androgen receptor positivity can increase the risk for hemorrhage.

Transdermal daily dosing has a relatively steady release of testosterone from a subdermal reservoir.^13^ In contrast, the pharmacokinetics of injections demonstrate significant peaks and troughs of serum testosterone.^7^ Therefore, to lower the risk for adenoma recurrence, transdermal daily dosing was recommended over parenteral injection for this patient.

To conclude, hepatic adenomatosis in TGD individuals on GAHT is uncommon and may present diverse molecular patterns and clinical characteristics not specific to sex assigned at birth or type of exogenous hormone. Treatment should rely on the pathology molecular profile, clinical features, and patient preferences. In cases of hepatic adenomatosis that develop on GAHT, pathology confirming a low risk for malignant transformation can circumvent the need for invasive procedures. Furthermore, reinitiating GAHT should consider ways to minimize risk and improve quality of life.

## DISCLOSURES

Author contributions: Conception: L. Yang. Initial draft: Y. Huang. Data collection: All authors. Key edits: Y. Huang, L. Yang, AY Chang and NM Loo. All authors edited and approved the final manuscript submitted. Y. Huang is the article guarantor.

Financial disclosure: None to report.

Informed patient consent was obtained for this case report.
